# *Archaeopteryx* feather sheaths reveal sequential center-out flight-related molting strategy

**DOI:** 10.1038/s42003-020-01467-2

**Published:** 2020-12-08

**Authors:** Thomas G. Kaye, Michael Pittman, William R. Wahl

**Affiliations:** 1 Foundation for Scientific Advancement, Sierra Vista, AZ 85650 USA; 2 Vertebrate Palaeontology Laboratory, Division of Earth and Planetary Science, The University of Hong Kong, Pokfulam, Hong Kong SAR China; 3 Wyoming Dinosaur Center, Thermopolis, WY 82443 USA

**Keywords:** Evolution, Palaeontology

## Abstract

Modern flying birds molt to replace old and worn feathers that inhibit flight performance, but its origins are unclear. We address this by presenting and evaluating a ~150 million year old record of molting in a feathered dinosaur from the early bird *Archaeopteryx*. Laser-Stimulated Fluorescence revealed feather sheaths that are otherwise invisible under white light. These are separated by one feather and are not in numerical sequential order and are mirrored in both wings. This indicates that a sequential center-out molting strategy was already present at the origins of flight, which is used in living falcons to preserve maximum flight performance. This strategy would have been a welcome advantage for early theropod flyers that had poor flight capabilities. This discovery provides important insights into how birds refined their early flight capabilities before the appearance of the keeled sternum, pygostyle and triosseal canal.

## Abstract (web summary)

Thomas Kaye et al. use Laser-Stimulated Fluorescence and fossil evidence from the oldest known bird, *Archaeopteryx*, to document the oldest record of molting, demonstrating that a sophisticated molting strategy developed unexpectedly early on in the evolution of avian flight. This discovery provides important insights into the flight capabilities of the earliest birds and predates other major flight adaptations.

## Introduction

Modern flying birds molt in a variety of ways with the goal of replacing old and worn feathers that inhibit flight performance^1,2^. Molting is often constrained by feather growth because its rate has a narrow range that does not scale with feather size^3^. Feather growth rates for smaller or larger birds are typically between 3.2 and 6.3 mm per day for primary feathers^3^. For smaller birds with correspondingly smaller feathers, the remiges can grow out in a single season, leading to complete replacement^3^. Larger birds tend toward partial molts known as *Staffelmauser* so complete replacement extends over several seasons^3,4^.

The most common molting strategy is a sequential molt, where feathers are lost from the left and right wing at the same time and proceed in a symmetrical pattern^4^. The sequence of feather loss itself has two strategies. The first strategy is a numerically sequential molt where feathers are lost in numerical order and is the most common among passerines. Typically, the primary feather molt proceeds from the innermost feather sequentially outwards to the tip^3^. The second strategy is a center-out approach where a center feather is lost first and then subsequent feathers are shed outwards from this center point; this is more common in non-passerine birds such as falcons^5^. This minimizes the size of the aerodynamic hole in the wing, which allows falcons to better maintain their flight performance for hunting^3,6^.

Pennaraptorans are the group of theropod dinosaurs that generically have vaned feathers and include birds^7^. They also comprise oviraptorosaurians and the closest bird relatives, the dromaeosaurids and troodontids. We define birds in this study as Avialae Gauthier 1986, a stem-based taxon containing *Passer domesticus* Linnaeus, 1758, and all coelurosaurian theropods closer to it than to *Dromaeosaurus albertensis* Matthew and Brown, 1922, or *Troodon formosus* Leidy, 1856 (*sensu*^7–9^). The ancestral feathered pennaraptoran did not fly^7^, so its feathers would not have molted due to flight performance pressure and presumably did so non-systematically. Among early fossil pennaraptorans, molting has only been identified in the Early Cretaceous flying dromaeosaurid *Microraptor*^10,11^. The authors observed a sequential molt strategy, but did not explicitly state which specific sequential strategy was involved: numerical or center out. The authors based this interpretation on the feather gap in the wing of specimen IVPP V13352 and three primary feathers that decrease in length outwards^10^. As the feathers are changing length in a numerical sequence, this suggests that *Microraptor* adopted a numerically sequential molting strategy. The sequential molt in *Microraptor* and molting data in extant birds was used by Kiat et al.^10^ to suggest that sequential molting is the outcome of evolutionary forces to maintain flight capability throughout the entire annual cycle in both extant birds and non-avialan paravian theropods^10^. Although it was not stated in their study, the ancestral molting condition for Paraves is presumably numerically sequential molting.

*Archaeopteryx* from the Late Jurassic Solnhofen Limestones of southern Germany is currently considered the oldest undisputed fossil bird and is iconic in our understanding of avian origins and of evolution more generally^12^. The Thermopolis specimen of *Archaeopteryx* WDC-CSG-100 (Wyoming Dinosaur Center)^13,14^ was scanned under Laser-Stimulated Fluorescence (LSF)^15^ (see Methods). This revealed anatomy not seen in normal light. In this study we identify the earliest evidence of pennaraptoran feather molting to our knowledge and discuss its implications on the origins of flight-related molting and of flight.

## Results and discussion

**Fig. 1:**
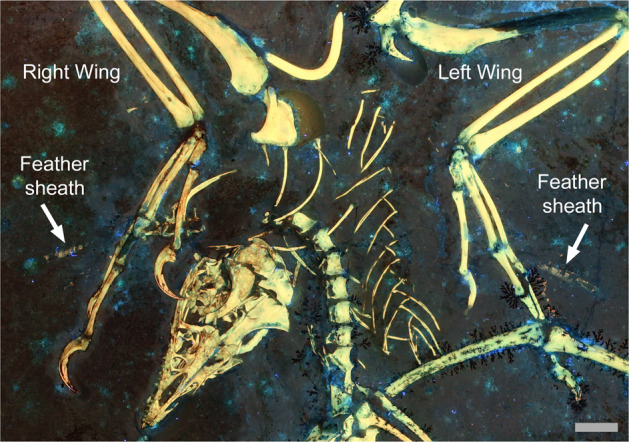
Bilaterally symmetrical cornified feather sheaths on the wings of *Archaeopteryx* under LSF. Molting feathers matched on both wings of WDC-CSG-100 ensured balanced aerodynamic performance. The white arrows indicate the feather sheaths. Scale bar is 1 cm.

WDC-CSG-100 exhibited matching elements on both wings under Laser-Stimulated Fluorescence (LSF) imaging (Fig. 1). These elements were found at the proximal ends of the rachises of the middle primary feathers. Unlike the absence of a UV or LSF reaction by the feather calami in the Berlin (MB.Av.101), Eichstätt (JME 2257), Solnhofen (BMMS 500), Munich (BSP 1999 I 50), and eleventh (no. 02923) specimens of *Archaeopteryx*^12,16–19^, the reaction of the matched elements in WDC-CSG-100 under LSF (Fig. 1) rejects the hypothesis that the elements are unusually thickened feather calami made of keratin.

Cornified feather sheaths encase the proximal end of new feathers during the growth phase, which fits the shape and position of the matching elements. The feather follicle embedded in the dermis, initially surrounds the developing feather filament^20^. The center of the filament contains the blood supply that feeds the growing feather^20^. The outer filament has three layers, the inner two form the feather proper while the outer most layer forms a keratinous sheath^20^. Once the feather has finished growing, the sheath dries up and falls off or is preened out. Thus, the matching elements could be molting feather sheaths.

**Fig. 2:**
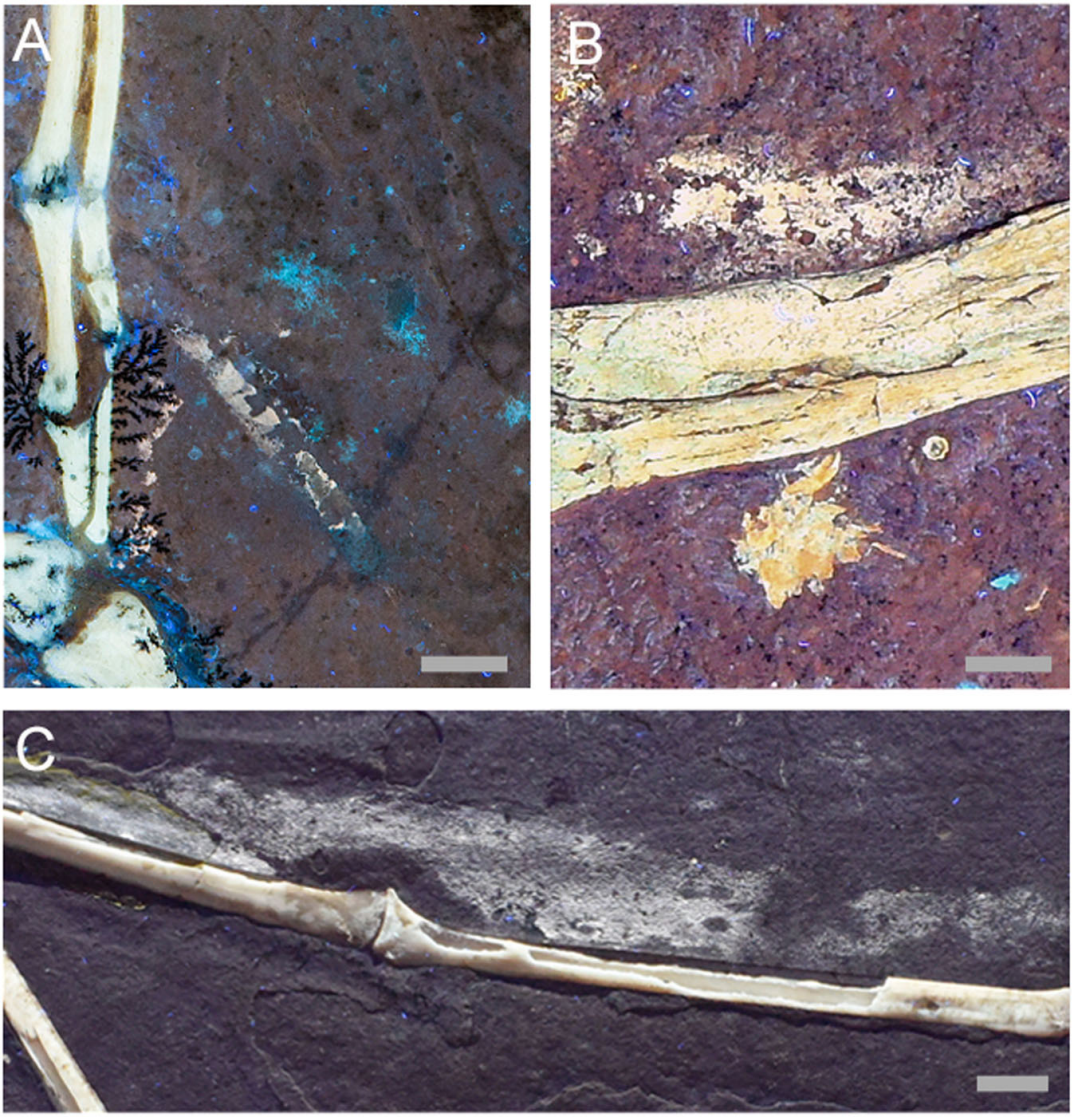
Comparison of preserved organic tissues in Solnhofen fossils. **a** Feather sheath from WDC-CSG-100. **b** Skin preservation on *Juravenator* JME Sch 200 (Jura Museum Eichstätt). **c** Wing membrane preserved in an undescribed pterosaur from the Jura Museum Eichstätt (JME SOS 2016). Scale bars: **a**, 0.5 cm, **b** 4 mm, and **c** 1 cm.

The remaining possibility for stick-like elements in the same part of the pennaraptoran wing are bony extensions like the ‘styliform’ elements of the membrane-winged scansoriopterygids^21,22^. However, examination of the calcium elemental map produced from the SLAC National Accelerator Laboratory beamline^23^ (Fig. 2) showed a lack of calcium in these elements which rules this hypothesis out.

Remnant elemental signatures of skin are uncommon in the Solnhofen fossil record but examples such as the non-avialan theropod *Juravenator* and several pterosaurs (Fig. 2) show such signatures under LSF and ultra violet (UV) light^24,25^. The matching elements fluoresce like preserved skin in other Solnhofen fossils indicating that they are not bone. This supports the remaining hypothesis that the matching elements are epithelial feather sheaths made up of cornified cellular tissue^26^. This is also congruent with the lack of calcium in the SLAC elemental analysis^23^ (Fig. 2). Cornified tissue was previously detected under LSF with a similar fluorescence signature in the tail bristles of the ornithischian dinosaur *Psittacosaurus*^27,28^.

**Fig. 3:**
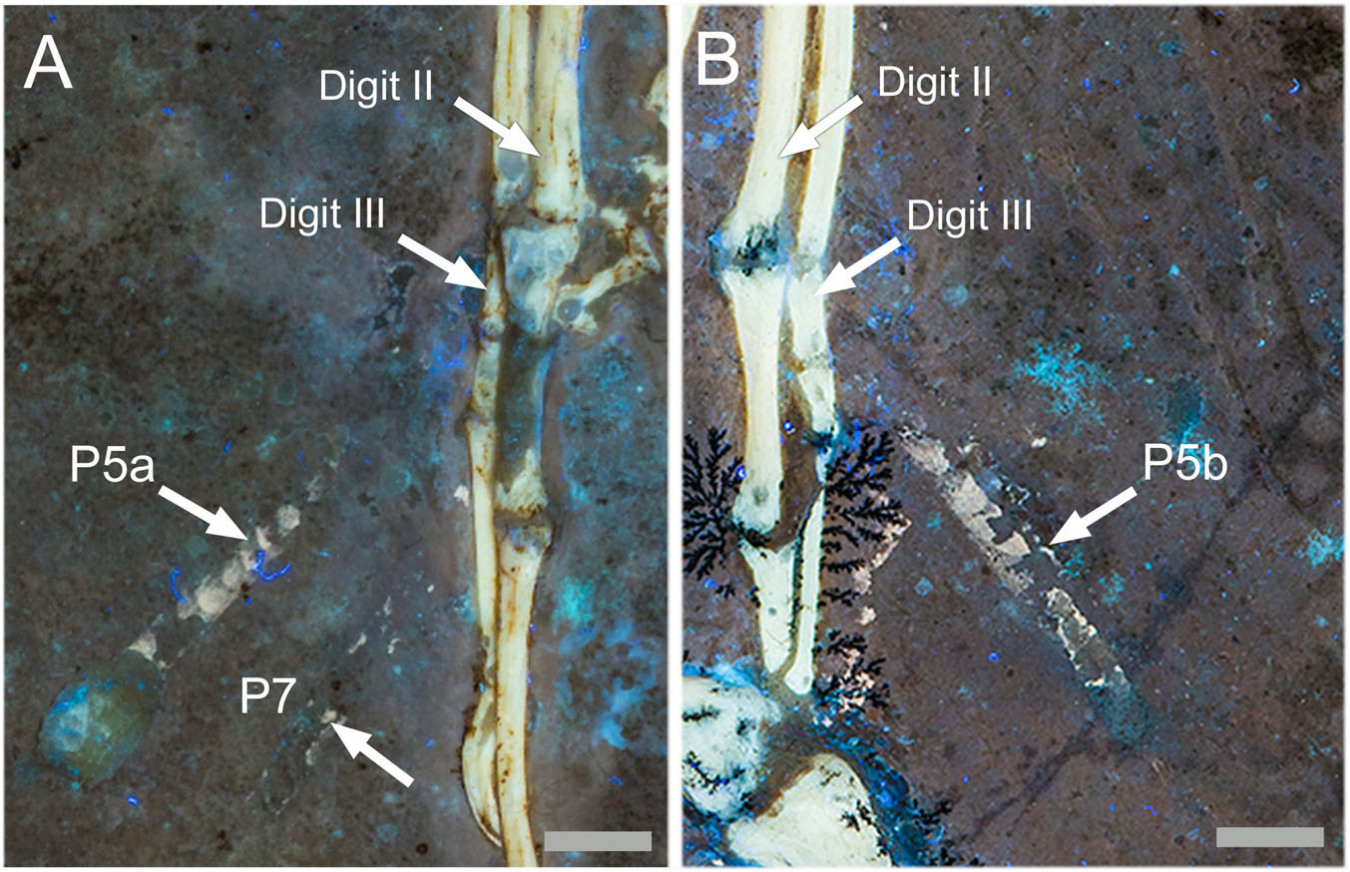
Close up of the hands of WDC-CSG-100 under LSF showing the position of the bilaterally symmetrical cornified feather sheaths. **a** Arrow P5a is the feather sheath on the fifth right primary feather. Arrow P7 shows remnants of the third sheath on the seventh primary feather. The sixth primary feather has already molted out and the molt sequence is proceeding in two opposite directions. **b** Arrow P5b points to the feather sheath on the fifth left primary feather which matches the one preserved on the right hand (arrow P5a). Scale bars are 0.5 cm.

Figure 3 shows the two wings of WDC-CSG-100 side by side demonstrating paired symmetry of the fifth primaries. In Fig. 3a, arrow P7 identifies the remnant of a third sheath that is not paired on the opposite wing. These elements are not visible on the slab under white light or on other *Archaeopteryx* specimens imaged using LSF. The two elements are bilaterally symmetrical as would be expected from what is observed in modern molting pairs. The elements exhibit matching widths and lengths and stop short of contacting the bone as would be expected if they were anchored into the patagia as in modern birds. The sheaths center on the axis of the rachis as shown in the phosphorous distribution map from the Bergmann et al.^23^ SLAC image (Fig. 1). This sheath alignment rules out their association with molting primary covert feathers.

**Fig. 4:**
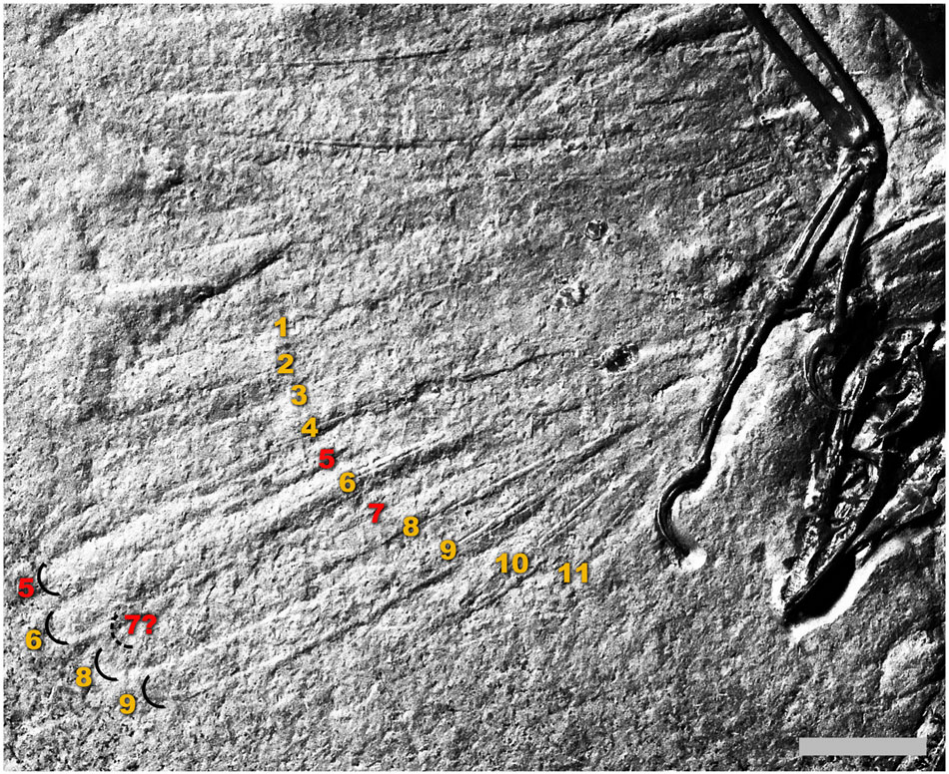
Right wing of WDC-CSG-100 under white raking light showing numbered primary feathers. Arrow delimiting the full length of a molted feather in WDC-CSG-100. This figure shows that primaries #5 and #7 were nearly full length. Cornified feather sheaths on primaries #5 and #7 are visible under LSF in Fig. 3a. The numbering on the feathers follows Mayr et al.^13^. The image is in grayscale and under white raking light to make the feathers easier to see. Scale bar is 2 cm.

The feather outlines on the right wing shows that the feathers were grown out and the feather sheaths were starting the process of being shed (Fig. 4). The primary count of 11^13^ suggests that a sequential “center out” molting strategy would start with either the fifth or sixth primary feather as the central vane from which to start the molt. The detection of feather sheaths one feather apart, suggests that the molt started with the sixth primary feather and proceeded out to the fifth and seventh which preserve the structures. This matches the modern sequential “center out” molting strategy that maintains maximum flight potential^6^. The symmetrical loss of the primary feathers balances the wing aerodynamics during the molt and maintains the best possible control when dealing with aerodynamically degraded wings^6^.

## Conclusions

The sequential “center out” molting strategy described here in *Archaeopteryx* is at least 150 million years old. To the best of our knowledge, this discovery is the oldest record of pennaraptoran feather molting (~25 million years older than the sequential molting record in *Microraptor*^10^) and the only record of adult molting in an early fossil bird (at least 20 million years older than existing avialan records from juvenile specimens from Spain and China^29^). Vaned feathers first evolved in pennaraptoran dinosaurs that could not fly^17^. This included asymmetrically vaned feathers that were traditionally associated with flight capability^30^. Thus, there was no original evolutionary pressure for a specific type of flight-related molting strategy, so molting presumably happened in a non-systematic manner early on. Asymmetrically vaned feathers were rapidly co-opted into the wings of early birds for powered flight^30^. A molting strategy that could maintain flight performance would have been a welcome advantage for early theropod flyers which had relatively poor flight capabilities^11^. Among early fossil pennaraptorans, numerically sequential molting has only been found in non-avialan pennaraptorans^10^. However, its prevalence among modern birds^10^ suggests that it was probably the ancestral condition of flying pennaraptorans. This discovery of a flight-performance-maintaining center out molting strategy in the Late Jurassic suggests the presence of two sequential molting strategies in the earliest pennaraptorans soon after the presumably non-systematic molting of the flightless pennaraptoran ancestor. This suggests that the evolutionary forces affecting the earliest pennaraptoran fliers included the optimization of molting strategies to benefit flight performance. Understanding why these strategies predate later flight-related innovations like the keeled sternum, pygostyle, and triosseal canal^31–35^ would be important future priorities and underscores the complexity of flight-related evolutionary changes along the lineage to modern birds.

## Methods

*Archaeopteryx* specimen WDC-CSG-100 – known colloquially as the ‘Thermopolis specimen’ – is housed at the Wyoming Dinosaur Center (Thermopolis, Wyoming, USA) where it is on permanent display to the general public and available to qualified researchers for academic study^14^. WDC-CSG-100 was photographed under white light and LSF. The camera was a Nikon D810 with a Sigma 35-mm lens. A 405 nanometer laser diode was projected through a line lens produced by *Laser Line Optics Canada* and scanned over the specimen according to the protocol outlined in Kaye et al.^15^ and extended in Wang et al.^36^. Color was equalized in the images using *Photoshop CS6* to ensure that the fluorescence signal was fully documented and analyzed.

### Reporting summary

Further information on research design is available in the Nature Research Reporting Summary linked to this article.

## Supplementary information

Reporting Summary

## Data Availability

All the data associated with this paper is available in full in the paper. The specimen WDC-CSG-100 is housed at the Wyoming Dinosaur Center, Thermopolis, Wyoming, USA.
